# Intracranial vasculopathy: an important organ damage in young adult patients with late-onset Pompe disease

**DOI:** 10.1186/s13023-024-03282-y

**Published:** 2024-07-15

**Authors:** Yuying Zhao, Xiaolin Yu, Duoling Li, Jingzhen He, Yuzhi Li, Bin Zhang, Na Zhang, Qian Wang, Chuanzhu Yan

**Affiliations:** 1 Research Institute of Neuromuscular and Neurodegenerative Diseases and Department of Neurology, Qilu Hospital, Cheeloo College of Medicine, Shandong University, Jinan, 250012 China; 2 Department of Geriatric Medicine, Qilu Hospital, Cheeloo College of Medicine, Shandong University, Jinan, 250012 China; 3 Department of Radiology, Qilu Hospital, Cheeloo College of Medicine, Shandong University, Jinan, 250012 China; 4 Department of Neurology, Jining NO.1 People’s Hospital, Jining, 272002 China; 5 Department of Neurology, Liaocheng People’s Hospital, Liaocheng, 252000 China; 6 Mitochondrial Medicine Laboratory, Qilu Hospital (Qingdao), Shandong University, Qingdao, 266035 China; 7 Brain Science Research Institute, Shandong University, Jinan, 250012 China

**Keywords:** Late-onset Pompe disease, Stroke, Vertebrobasilar dolichoectasia, Arterial stenosis, Cerebral small vessel disease

## Abstract

**Background:**

Late-onset Pompe disease (LOPD) is mainly characterized by progressive limb-girdle muscle weakness and respiratory impairment, whereas stroke and cerebrovascular abnormalities have been insufficiently studied in LOPD. This study aimed to evaluate the frequency and pattern of intracranial artery and brain parenchyma abnormalities in LOPD patients.

**Results:**

Neuroimaging data from 30 Chinese adult LOPD patients were collected from our center. Seven patients (7/30) had acute cerebral infarction or hemorrhage. Brain magnetic resonance angiography (MRA) or computed tomography angiography (CTA) revealed artery abnormalities in 23 patients (23/30). Dilative arteriopathy was found in 19 patients (19/30), with vertebrobasilar dolichoectasia found in 17 patients and dilatation of the anterior circulation arteries found in 8 patients. The maximum diameter of the basilar artery was correlated with disease duration (*p* < 0.05). In addition, aneurysms (7/30) and fenestrations (3/30) were discovered. There were 14 patients with arterial stenosis (14/30), and both anterior and posterior circulation involvement occurred in 9 patients (9/14). Stenosis and dilative arteriopathy simultaneously occurred in 10 patients (10/30). White matter hyperintensities were present in 13 patients (13/28). Microbleeds, predominantly located in the cerebellum and brainstem, were detected in 7 patients (7/22) via susceptibility-weighted imaging.

**Conclusions:**

Intracranial vasculopathy involving both large arteries and small vessels is an important organ damage in LOPD patients. LOPD should be considered a key differential diagnosis in young adults with cryptogenic stroke, and a series of imaging evaluations of the brain and intracranial blood vessels is recommended as a routine workup in adult LOPD patients.

## Background

Pompe disease, also known as glycogen storage disease type II (OMIM# 232,300), is an autosomal recessive disorder caused by acid alpha-glucosidase (GAA) deficiency leading to excessive glycogen accumulation in lysosomes [1]. The skeletal, cardiac and respiratory muscles are more prone to be affected. Pompe disease has great genetic and clinical heterogeneity. Late-onset Pompe disease (LOPD) is characterized by progressive myopathy and respiratory insufficiency in childhood and adulthood [2]. The central nervous system could also be involved [2, 3]. Autopsy findings of the cerebral vasculature of individual LOPD patients revealed lysosomal glycogen accumulation in smooth muscle cells of the smaller arteries, arterioles and capillaries [4–7].

Cerebrovascular disease in LOPD patients has been predominantly reported in case reports [8–10]. Cerebral hemorrhage, subarachnoid hemorrhage, aneurysm and dilation of vertebrobasilar arteries were most frequently researched [8, 11–13]. However, cerebral infarction, artery stenosis and cerebral small vessel disease (CSVD) in LOPD patients have seldom been reported [9, 14]. Most neuroimaging studies on LOPD patients in the literature were from European countries, with the largest studies including 21 patients [3, 13, 15, 16]. However, only case reports or family studies on cerebral vessel involvement in Asian LOPD patients exist [9, 17]. Herein, we collected neuroimaging data from Chinese adult LOPD patients at our center and evaluated their cerebrovascular and brain parenchyma abnormalities.

## Methods

### Identification of subjects

We retrospectively investigated LOPD patients diagnosed at our center between November 2009 and March 2023. The diagnosis was confirmed by a GAA assay, muscle biopsy and genetic mutation analysis. Although aged > 1 year at disease onset, the patients were first diagnosed as adults. All the patients underwent neuroimaging evaluation of their brain parenchyma and cerebral vessels, and the patients were aged ≥ 18 years. Patients were excluded if the imaging data were incomplete or if their age at the time of imaging was less than 18 years. In total, 30 Chinese patients (ranging from 18 to 61 years, median age 28.5 (8.5) years) were included, including 14 males and 16 females (Table 1). Since 26 LOPD patients underwent brain magnetic resonance angiography (MRA) scans, 26 age- and sex-matched healthy controls were enrolled. This study was approved by the Ethics Committee of Qilu Hospital.

**Table 1:** Clinical features of 30 adult LOPD patients

Pt	Sex	Imaging age (y)	Disease duration (y)	Initial symptom	GAA gene mutation	
1	M	20	12	LGMW	c.546G > A	c.2662G > T
2	M	28	7	Dyspnea during sleep	c.2238G > C	c.2238G > C
3	M	52	27	Lower extremities ache after exercise	c.1562 A > T	-7G > A
4	F	25	4	LGMW	c.546G > T	c.1735G > A
5	M	27	11	LGMW	c.2105G > A	c.2238G > C
6	M	19	6	LGMW	c.837G > C	c.2238G > C
7	F	31	7	Respiratory failure	c.1669 A > T	c.2132 C > G
8	F	45	16	LGMW	c.1822 C > T	c.2240G > A
9	F	42	2	LGMW	c.1822 C > T	c.2240G > A
10	F	30	4	LGMW	c.2105G > A	c.2238G > C
11	M	32	17	LGMW	c.2105G > A	c.2238G > C
12	F	31	24	LGMW	-32-13T > G	c.1839G > A
13	M	19	6	LGMW	c.796 C > T	c.1634 C > T
14	F	19	2	Dyspnea after exercise	c.784G > A	c.2238G > C
15	M	61	21	LGMW	c.2173 C > T	c.2173 C > T
16	M	28	16	LGMW	c.2040G > T	c.2238G > C
17	F	27	26	LGMW	c.1222 A > G	c.2238G > C
18	F	32	10	LGMW	c.1871_1872del	c.2237G > A
19	F	18	3	LGMW	c.241 C > T	c.1309 C > T
20	F	30	0	Stroke	c.1309 C > T	c.1309 C > T
21	F	52	10	LGMW	c.-32-13T > G	c.503G > C
22	M	32	12	LGMW	c.796 C > T	c.2238G > C
23	M	18	9	LGMW	c.1280T > C	c.2238G > C
24	M	18	5	LGMW	c.1562 A > T	c.2238G > C
25	F	40	13	LGMW	c.241 C > T	c.2238G > C
26	F	33	10	LGMW	c.241 C > T	c.2238G > C
27	M	29	12	LGMW	c.241 C > T	c.2238G > C
28	M	28	14	LGMW	c.1388_1406del	c.2238G > C
29	F	26	8	Stroke	c.1388_1406del	c.2238G > C
30	F	28	15	LGMW	c.827_845del	c.2238G > C

*F* female, *LGMW* limb girdle muscle weakness, *M* male

### Scan protocol and image analysis

A total of 28 LOPD patients underwent routine brain magnetic resonance imaging (MRI) and diffuse weighted imaging (DWI) examinations; the other two underwent computed tomography (CT) scans as an emergency (Table 2). Images were acquired on a Siemens Verio 3.0 Tesla MRI scanner (Siemens, Erlangen, Germany). Twenty-two LOPD patients had susceptibility-weighted imaging (SWI) scans. Brain time-of-flight MRA was completed in 26 out of the 28 patients via MRI. The remaining 2 patients with MRI data and the 2 patients with CT data underwent brain CT angiography (CTA) with a 256-slice spiral CT scanner (Brilliance iCT, Philips Healthcare, Netherland) (Table 2). SWI was not conducted in the healthy controls.

**Table 2:** Neuroimaging characteristics of 30 adult LOPD patients

Pt	Artery dilatation/aneurysm (An.)/fenestration (Fe.)	Artery stenosis	Stroke lesion	Fazekas score	Microbleeds
1	0	0	0	0	NA
2	BA & VA ectasia, MCA	0	0	0	0
3	0	0	Left brachium pontis	2	0
4	ICA	0	0	0	0
5	VD, ICA, BA (An.), RACA (An.), RMCA (Fe.)	0	0	3	NA
6	BD, VA ectasia, ICA	0	0	0	NA
7	ICA, RMCA (Fe.)	Bi. ACA, MCA & PCA, LVA	Right BG	4	BS & bi. cerebellum
8	0	0	0	2	0
9	0	0	0	0	0
10	BA (Fe.)	LMCA, RPCA	0	3	NA
11	BD, VA elongation	LMCA, LVA	0	0	0
12	BA & VA ectasia, ICA, LMCA (An.)	0	0	0	0
13	VA elongation	0	0	0	0
14	0	LACA, RMCA	0	0	0
15	VA elongation	0	0	2	Cerebellum
16	VA elongation, BA (An.)	Bi. MCA, RVA	0	0	0
17	VA elongation, RMCA (An.)	Bi. ACA, MCA & ICA	0	2	0
18	VA tortuosity	RACA, LMCA	0	0	0
19	VA elongation, ICA	RACA, bi. MCA, LVA	0	4	Medulla oblongata
20	VA elongation	Bi. ACA & PCA, RMCA, BA	Bi. occipital & right parietal lobes, right BG & cerebellum	0	NA
21	BD, VA elongation & tortuosity	Bi. ICA	0	0	0
22	VD, MCA	0	0	1	Temporal-occipital area
23	0	0	0	0	0
24	0	0	0	0	NA
25	0	RICA, RVA	Right cerebellum	3	0
26	BA (An.)	Bi. ACA & PCA	Right brachium pontis	4	BS & bi. cerebellum
27*	0	0	0	NA	NA
28^†^	BA ectasia, BA (An.), RVA (An.)	Bi. ACA & MCA, RPCA	Left cerebellum	4	BS & cerebellum
29^†^	BA ectasia	Bi. PCA	BS hematoma	5	BS, bi. cerebral hemisphere, thalamus & cerebellum
30*	BD, BA (An.)	0	0	NA	NA

*ACA* anterior cerebral artery, *An.* aneurysm, *BA* basilar artery, *BD* BA dolichoectasia, *BG* basal ganglia, *Bi.*/*bi.* Bilateral/bilateral, *BS* brain stem, *Fe.* fenestration, *ICA* internal carotid artery, *L* left, *MCA* middle cerebral artery, *NA* not available, *PCA* posterior cerebral artery, *R* right, *VA* vertebral artery, *VD* VA dolichoectasia * Pt27 and Pt30 had computed tomography (CT) and CT angiography (CTA) scans

^†^ Pt28 and Pt29 had magnetic resonance imaging and CTA scans

The MRA image data were transferred to a Siemens Syngo workstation. Two experienced neuroradiologists independently detected intracranial aneurysms, arterial stenosis and dilation. Vertebrobasilar dolichoectasia (VBD) was diagnosed according to the criteria proposed by Ubogu and Zaidat [18]: a basilar artery (BA) or a vertebral artery (VA) diameter > 4.5 mm or deviation of any portion that is > 10 mm from the shortest expected course, a BA length > 29.5 mm or an intracranial VA length > 23.5 mm. The diameters of the distal internal carotid artery (ICA), middle cerebral artery (MCA) and anterior cerebral artery (ACA) were measured as described elsewhere [19]. Dilation of the anterior circulation artery was considered when the diameter was above three times the standard deviation (SD) of the control values [13]. Intracranial artery stenosis was defined as any degree of stenosis in at least one of the above arteries and in the posterior cerebral artery (PCA) [20].

The volumetric CTA data were transferred to the workstation, and the images were analyzed by the aforementioned neuroradiologists. Postprocessing, including multiplannar reformation, maximum intensity projection and volume rendering reformation, was completed. According to the Smoker’s criteria [21], BA ectasia was defined as a diameter > 4.5 mm; “dolicho” was added when the BA was laid lateral to the margin of the clivus or dorsum sellae or when the BA bifurcation was located above the suprasellar cistern plan.

Cerebral hemorrhage and infarct lesions were traced. White matter hyperintensities (WMHs) were analyzed on T2-fluid-attenuated inversion-recovery (T2-FLAIR) images and rated with the Fazekas scale [22, 23]. The total score was calculated by adding the scores for periventricular and deep white matter hyperintensity. Among the patients scanned with SWI, microbleeds were detected.

### Statistical analysis

All the statistical analyses were performed using SPSS version 22.0 (SPSS, Inc., International Business Machines, Chicago, Illinois, USA). The mean ± SD or median and interquartile range were used for continuous variables in the descriptive analysis. Unpaired Student’s *t* test and the Mann‒Whitney test were used to assess differences between two groups for continuous variables. Spearman’s rho and Fisher’s exact tests were used for the correlation analysis of continuous and categorical variables, respectively. We applied binary logistic regression and ordinal regression to analyze risk factors for stroke and WMH. A *p* value < 0.05 was considered statistically significant.

## Results

### Clinical data analysis

The most common symptom in the 30 adult LOPD patients was muscle weakness, especially in the lower limb-girdle muscles (Table 1). Respiratory impairment was another predominant symptom. The central nervous system involvement included dizziness, hypodynamia, hemiplegia and walking instability due to cerebral infarction or hemorrhage. Seven patients had acute ischemic or hemorrhagic stroke during the disease (7/30), and stroke was the initial attack in 2 patients. Common cardiovascular risk factors, such as obesity, smoking status and alcohol consumption, were not present in our patients, except for diabetes mellitus (*n* = 1), hypertension (*n* = 1) and hyperlipidemia (*n* = 1). Nine patients were from 4 pedigrees. Only one had a biopsy if another patient was a sibling. Muscle biopsy was performed for 25 patients. Histopathology revealed vacuolar myopathy with basophilic granule aggregation. All patients had less than 30% of the normal GAA enzyme activity. The most frequent GAA gene mutation was c.2238G > C (17/30).

### Dilative arteriopathy, aneurysm and fenestration

**Fig. 1:**
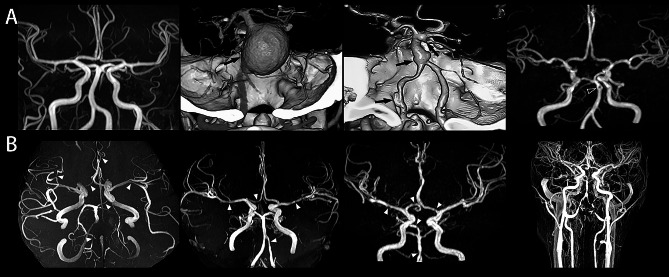
Cerebral artery abnormalities in adult LOPD patients. (**A**) Dilative arteriopathy, aneurysms (arrows) and fenestration (hollow arrowhead) were revealed. (**B**) Magnetic resonance angiography showed multiple stenoses of cerebral arteries (arrowheads) and bilateral vertebral artery stenosis on neck angiography (angles)

Brain MRA and CTA revealed that 23 patients had intracranial arteriopathy (23/30) (Fig. 1). Together, 21 patients had cerebral artery dilation, aneurysm, or fenestration (21/30) (Table 2). VBD was discovered in 17 patients (17/30), with BA dolichoectasia in 4 patients, VA dolichoectasia in 2 patients, ectasia of BA or VA without “dolicho” in 4 patients, and “dolicho” of VA without BA involvement in 7 patients. The maximum diameter of the BA was correlated with disease duration (Spearman rho = 0.519, *p* = 0.007) but not with age at imaging. Compared with that in the controls, the distal ICA in LOPD patients was enlarged on MRA; however, the diameters of the MCA and ACA were not significantly different (Table 3). Dilative arteries in the anterior circulation, the diameters of which were above 3 times the SD of control values, were detected in 8 patients; these included dilation of the distal ICA in 6 patients and of the MCA in 2 patients (Tables 2 and 3). There was a statistically insignificant correlation between the diameter of the anterior circulation arteries and imaging age or disease duration. Without regard for aneurysm or fenestration, 19 patients had dilative arteriopathy, among whom 6 had both anterior and posterior circulation involvement and 2 had solo dilation of the ICA in the anterior circulation. The genetic-radiological correlation could not be calculated. The genotypes of our patients were variable, and a few patients had a variant of uncertain significance on one allele. In addition, patients from a family with the same GAA genotype had different neuroimaging results.

**Table 3:** Diameters of anterior circulation arteries in adult LOPD patients and healthy controls

Variable	LOPD patients (*n* = 26)	Controls (*n* = 26)	*P* value
Age of imaging	30.0 (15.0)	30.5 (13.8)	0.89*
Diameter of ACA	2.3 ± 0.5	2.3 ± 0.3 (3.2)	0.599^†^
Diameter of MCA	2.9 ± 0.5	2.8 ± 0.3 (3.6)	0.339^†^
Diameter of ICA	4.0 ± 0.7	3.4 ± 0.4 (4.5)	0.000^†^

*ACA* anterior cerebral artery, *ICA* internal carotid artery, *MCA* middle cerebral artery

* Values were analyzed by the Mann‒Whitney U test

^†^ Values were analyzed by unpaired Student’s *t* test

Aneurysm and/or fenestration was found in 9 patients. Seven patients had a total of 10 aneurysms on imaging. Notably, 7 of these aneurysms were located at the BA (*n* = 6) or the VA (*n* = 1), while the remaining 3 were situated in the anterior circulation. Additionally, 3 patients exhibited fenestration variants—one at the BA and the other two at the MCA. Only 2 out of the 9 patients did not have concurrent dilative arteriopathy. The healthy controls had no aneurysm or fenestration.

### Cerebral artery stenosis

There were 14 patients with cerebral artery stenosis in our cohort (14/30), and most of these patients were female (11/14). From the perspective of stenosis distribution, 47 branches of stenotic arteries were detected, including 31 branches in the anterior circulation (15 of the MCA) and 16 branches in the posterior circulation (10 of the PCA). Nine patients had arterial stenosis located in both the anterior and posterior circulation (9/14). Ten out of the 14 patients had arterial stenosis simultaneously combined with arterial dilation with or without aneurysm or fenestration (10/14). No cerebral artery stenosis was found in the 26 healthy controls.

### Stroke lesions

**Fig. 2:**
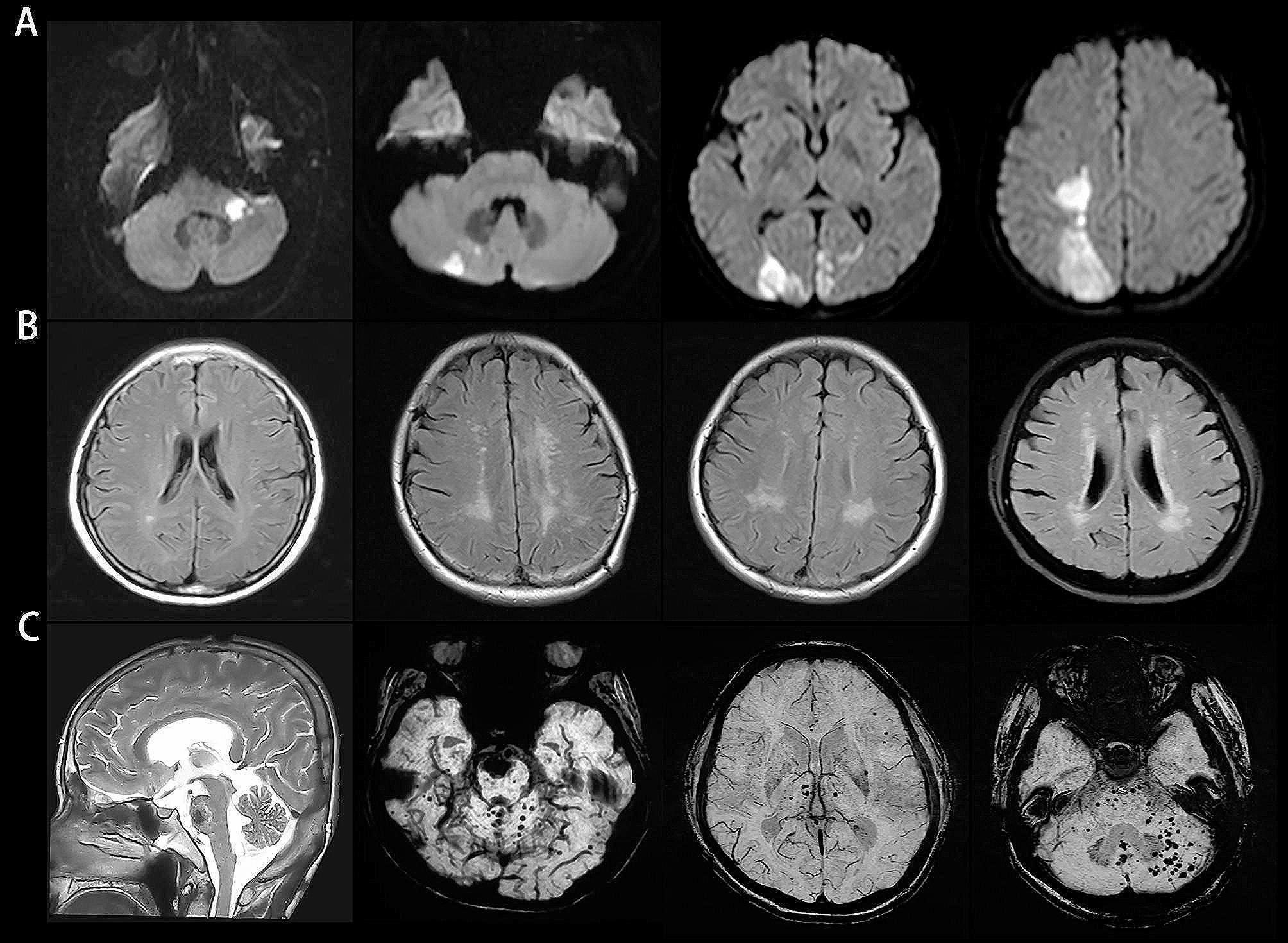
Brain parenchyma abnormalities in adult LOPD patients. (**A**) The infarct lesions were located at the brachium pontis, cerebellum and cerebral hemisphere on diffuse weighted imaging. (**B**) T2-fluid-attenuated inversion-recovery images showed white matter hyperintensities. (**C**) Hematoma in the brainstem and microbleeds in the brainstem, cerebellum, cerebral hemisphere and thalamus were detected via T2-weighted imaging and susceptibility-weighted imaging

Six patients had definite cerebral infarction, and one patient had cerebral hemorrhage on MRI and CT (Fig. 2). Specifically, the infarct and hematoma lesions were subtentorial in 5 patients, supratentorial in 1 patient, and both subtentorial and supratentorial in 1 patient (Table 2). All but one of the 7 patients with cerebrovascular events had cerebral arteriopathy or arterial stenosis. Among them, 5 also had arterial dilation, elongation or aneurysm. Arterial stenosis was a significant risk factor for stroke (odds ratio, 16.754; 95% confidence interval, 1.154-243.201; *p* = 0.039). No significant correlation was found between stroke incidence and age, sex, disease duration or artery dilatation. Stroke lesion was absent in the controls.

### Lesions of cerebral small vessels

T2-FLAIR images revealed WMHs in 13 patients (13/28). The Fazekas scores were 1 (1/13), 2 (4/13), 3 (3/13), 4 (4/13), and 5 (1/13). Only 2 patients with WMHs had no large artery abnormalities; nevertheless, the remaining 11 patients all had cerebral arterial ectasia, elongation, aneurysm or fenestration, with or without stenosis. In addition, among the 13 patients, 6 with Fazekas scores ≥ 2 had stroke. No significant correlations were detected between WMH and age, sex or disease duration. WMHs were found in 2 healthy controls (Fazekas score = 1).

Microbleeds on SWI were found in 7 patients (7/22); furthermore, the cerebellum and/or brainstem were involved in 6 patients (6/7) and were even present in 4 out of the 7 patients with stroke. All patients with microbleeds had arterial dilation, elongation or aneurysm.

## Discussion

LOPD has a variety of clinical and genetic manifestations. In our group, the c.2238G > C mutation was identified as the most common. We investigated the neuroimaging characteristics of Chinese adult LOPD patients at our center. Most of the patients had progressive proximal muscle weakness and respiratory impairment. Seven patients had stroke. Enzyme assays, muscular histopathology and genetic mutation analysis confirmed the diagnosis. Cerebral arteriopathy and brain parenchyma abnormalities were detected.

For the cerebral vasculature in LOPD patients, dolichoectasia of the vertebrobasilar system was most frequently reported in the literature [13, 15, 24, 25]. In general, the incidence of VBD in the general population is 0.06–4.4% [16, 18]. However, in the study of 18 LOPD patients, Pichiecchio et al. discovered VBD using brain MRA or contrast-enhanced CT in 10 patients and no aneurysm in any of those patients [13]. Similarly, two studies from the same center involving 21 LOPD patients reported VBD in 10 or 11 patients and aneurysm in 2 or 3 patients by brain CTA or MRA, respectively [3, 15]. In our study, 17/30 patients had VBD, and 7/30 patients had aneurysms, 7/10 of which were located in the posterior circulation. The incidence of aneurysm (23.3%) was much greater than the 3.2% reported in the general population [26]. The mechanism of dilative arteriopathy or aneurysm in LOPD patients is not completely understood. Several autopsy studies have demonstrated that there is an abnormal accumulation of lysosomal glycogen within smooth muscle cells of cerebral arterioles and arteries in LOPD patients [4, 5, 24]. Excessive glycogen deposits can cause degeneration and necrosis of cells [5]. This process interferes with the synthesis and construction of the extracellular matrix and reduces the elasticity and integrity of the vessel wall [2, 19, 24], which may be correlated with dilative arteriopathy and aneurysm. Additionally, respiratory impairment increases the partial pressure of carbon dioxide in the blood, which may also lead to vasodilation. Compared to the anterior circulation, the posterior circulation has less sympathetic innervation, and the elastic layer is weaker, which predisposes patients to dilation or aneurysm. Aneurysm rupture can result in subarachnoid hemorrhage, intracranial hematoma or microbleeds, all of which are associated with poor prognosis [8, 17]. In our group, the diameter of the BA was correlated with disease duration but not with age at imaging, which is a finding that is not completely consistent with the findings in the literature [13] and might be due to the younger age at imaging in our patients. Arterial dilation in the anterior circulation was present in 8 of our 30 patients (8/30), including ectasia of the distal ICA in 6 patients and the MCA in 2 patients. Therefore, dilative arteriopathy may be more extensive than expected.

Intracranial artery fenestration was found in our 3 LOPD patients (10%), which was slightly greater than the frequency reported in a Chinese cohort study (6.2%) [27]. Fenestration is a rare anatomical variation that occurs during embryo development. Glycogen deposition may increase the vulnerability of the arterial wall, and fenestration may develop into an aneurysm due to hemodynamic stress and vessel wall fragility [28]. As a result, fenestration is more dangerous in LOPD patients than in the general population, and follow-up is necessary [15].

Notably, restrictive arteriopathy was also detected. Cerebral artery stenosis combined with ectasia/aneurysm/fenestration was more common than stenosis alone in our patients. The arterial stenosis involved multiple segments, not only the posterior circulation but also the anterior circulation. This phenomenon has rarely been reported in LOPD patients. Anneser et al. reported stenosis of the MCA and dilatation of the carotid artery and BA in one patient [29]. It was speculated that these abnormalities were related to the increase in arterial stiffness due to glycogen deposits in the artery wall and that the accumulation of these abnormalities was plurifocal and segmental [4]. Destruction of the intracranial arteriole wall initiates the formation of atherosclerotic plaques, which in turn leads to local stenosis or occlusion of the vessel, causing ischemic cerebrovascular disease [18]. Malhotra et al. reported a LOPD patient with initial symptoms of cerebellar and brainstem infarction due to vertebrobasilar artery stenosis, and the patient died of locked-in syndrome eight months later after rapid progression [14]. Arterial stenosis was a significant risk factor for stroke in our study. Hence, cerebral vessel stenosis can occur in adult LOPD patients, often with a poor prognosis. The involvement of arterial stenosis may be more diffuse than previously recognized. A study also showed an increase in aortic wall stiffness in Pompe disease patients [30].

Strokes in LOPD patients have been mainly reported as case reports/series [25]. Seven patients in our study had stroke. Some of our 30 patients underwent neuroimaging examinations after the occurrence of central nervous system symptoms. Hydrocephalus or compressive symptoms in the cranial nerve were absent in our patients. Only 3 of our patients had concomitant hypertension, hyperlipidemia or diabetes mellitus; i.e., cerebrovascular risk factors. It is supposed that the hematoma in the brainstem of one of our patients may be related to the rupture of a small aneurysm. Infarction lesions in the subtentorium appeared in 5 patients, including one patient with PCA territory and anterior circulation involvement; moreover, one patient had infarction lesion in the basal ganglion. The apparent intracranial artery stenosis was responsible for cerebral infarction in some patients. In addition, the infarct may be attributed to dilative arteriopathy, particularly VBD. VBD patients have a high incidence of posterior circulation infarction [18, 31]. Dilation, tortuosity and/or elongation of the vertebrobasilar artery was followed by hemodynamic changes. Reduced or stagnant blood flow and morphological features of arteries could result in intraluminal thrombosis in situ [9–11]. An embolus plugging the orifices of the perforating artery, embolus detachment, or hypoperfusion may contribute to cerebral infarction [11, 32].

To date, data regarding cerebral small vessels in LOPD patients are scarce. Nearly half of our patients (46%) had WMH, which was greater than the 26% of young clinical patients in another study [33]. Moreover, 11 patients with WMHs had coexistent large artery abnormalities. It was speculated that WMHs are caused by ischemia and insufficient cerebral oxygenation in LOPD patients [2, 3, 15]. Mellies et al. reported that LOPD patients developed respiratory disorders due to diaphragm weakness and inspiratory and upper respiratory tract muscle involvement, which caused a reduction in the cerebral blood flow and impaired tissue oxygenation [34]. The severity of WMH and the degree of large artery stenosis do not necessarily correlate with each other [35]. Emerging evidence has revealed that VBD or intracranial arterial dolichoectasia are related to small vessel disease [20]. No significant correlation was found between WMH and dilative arteriopathy in our study, possibly because of the small sample size. CSVD contributes to 25% of stroke cases in the general population [36]. However, 6 of the 7 patients with stroke had WMHs in our study. Hence, WMHs should also be considered in LOPD patients.

The presence of microbleeds is another brain MRI marker of CSVD. In the case study by Kretzschmar et al., glycogen-filled vacuoles were observed in arteries of different sizes, and hemosiderin was deposited around the vessels, which indicated a disorder of the blood‒brain barrier [5]. Moreover, numerous small aneurysms are also predominantly localized in the cerebellum [5]. SWI is sensitive for detecting intracranial chronic microbleeds, which present as multifocal black dots. In our study, we detected microbleeds in 7 LOPD patients (23.3%), which was higher than the incidence (10%) of microbleeds in 40-year-old or older healthy adults in the literature [37]. The lesions were located mostly in the cerebellum and brainstem, and this distribution was distinct from the distribution in amyloid angiopathy. Hemodynamic changes related to VBD or aneurysm may result in microbleeds in LOPD patients.

In young adult patients with stroke and in terms of genetic factors, Fabry disease has been the focus of research, while LOPD has been easily ignored. Our study revealed the complexity and diversity of cerebrovascular involvement in adult LOPD patients. However, our retrospective study has several limitations. The design was a single-center observational study. The sample size was small since the neuroimaging of many of our LOPD patients was absent or performed outside our institution. Two patients had an emergency CT rather than an MRI scan. Not every patient had a control due to failure to complete the MRA examination. We did not include respiratory parameters in our study, for not all of these parameters were acquired at the imaging time. In addition, follow-up neuroimaging and particular effects of enzyme replacement therapy on cerebral arteriopathy were lacking. Hensel et al. recommended that routine follow-up angiography be completed every 5 years in LOPD patients to monitor the dilated arteries [38]. Cerebral artery stenosis and brain parenchyma abnormalities should also be considered.

## Conclusion

The coexistence of artery dilation or dolichoectasia and stenosis, as well as anterior and posterior circulation involvement, is a feature of arteriopathy in our Chinese adult LOPD patients. In addition to those of the intracranial major arteries, small vessel changes revealed by WMHs and microbleeds also should not be ignored. LOPD should be considered in young adults with cryptogenic stroke. A series of imaging evaluations, including brain MRI, MRA and SWI, is recommended for adult LOPD patients.

## Data Availability

The datasets generated and analyzed during the current study are not publicly available due to the need to protect study participant privacy. The data are available from the corresponding author on reasonable request.

## Abbreviations

ACA: Anterior Cerebral Artery
BA: Basilar Artery
CTA: Computed Tomography Angiography
GAA: Acid Alpha-Glucosidase
ICA: Internal Carotid Artery
LOPD: Late-Onset Pompe Disease
MCA: Middle Cerebral Artery
MRA: Magnetic Resonance Angiography
MRI: Magnetic Resonance Imaging
PCA: Posterior Cerebral Artery
SWI: Susceptibility-Weighted Imaging
VA: Vertebral Artery
VBA: Vertebrobasilar Artery
WMHs: White Matter Hyperintensities
